# Prevalence, Antimicrobial Resistance Patterns, and Emerging Carbapenemase-Producing *Enterococcus* Species from Different Sources in Lagos, Nigeria

**DOI:** 10.3390/antibiotics14040398

**Published:** 2025-04-12

**Authors:** Wasiu Olawale Salami, Samuel Oluwasegun Ajoseh, Aminat Olajumoke Lawal-Sanni, Ashraf A. Abd El Tawab, Heinrich Neubauer, Gamal Wareth, Kabiru Olusegun Akinyemi

**Affiliations:** 1 Department of Microbiology, Faculty of Science, Lagos State University, Ojo P.O. Box 0001 LASU Post Office, Lagos 102101, Nigeria; wasiu.salami@lasu.edu.ng (W.O.S.); samuel.ajoseh@lasu.edu.ng (S.O.A.); aminat.lawal@lasu.edu.ng (A.O.L.-S.); 2 Department of Bacteriology, Immunology, and Mycology, Faculty of Veterinary Medicine, Benha University, Toukh 13511, Egypt; ashraf.awad@fvtm.bu.edu.eg; 3 Institute of Bacterial Infections and Zoonoses, Friedrich-Loeffler-Institut, 07743 Jena, Germany; heinrich.neubauer@fli.de

**Keywords:** carbapenemase, virulence, resistance, genes, *Enterococcus*, Nigeria

## Abstract

**Background**: *Enterococcus* species present significant health risks due to their widespread presence in humans, animals, and the environment. This study examined the patterns of antimicrobial resistance (AMR) and the presence of carbapenemase-producing *Enterococcus* species from various sources. **Methods**: Between November 2023 and February 2024, 500 samples were collected in Lagos State, including 350 clinical human samples, 50 environmental samples, and 100 animal samples. The samples were processed, and *Enterococcus* isolates were identified and subjected to antimicrobial susceptibility tests (AST) by standard methods. Furthermore, carbapenemase (*bla*_KPC_ and *oxa-48*) and virulence genes (*gelE*) were detected by real-time polymerase chain reaction (RT-PCR) methods using specific primers. **Results**: The overall prevalence of *Enterococcus* isolates was 4.6% (23/500), including 18 *E. faecalis* and 5 *E. faecium*. The source prevalence was 24% (12/50) from the environmental samples, 5% (5/100) from animal sources, and 1.7% (6/350) from the clinical samples. All *Enterococcus* isolates were 100% resistant to ciprofloxacin, erythromycin, imipenem, vancomycin, and ampicillin. However, 91% were susceptible to gentamicin. Six (6) distinct resistance profiles were observed, with the pattern AMP-ERY-TGC-CIP-TS-VA-CHL-AUG-MEM-IMI being the most frequent in 12 *E. faecalis* (4 isolates from humans, 2 from animals, and 6 from the environment). Notably, 39.1% (9/23) of multiple-drug resistant *Enterococcus* isolates harbored the *gelE* virulence gene, including seven *E. faecalis* (five environmental and two human) and two *E. faecium* from animal sources. The *E. faecalis* strains HB003 and HB050, from human bacteremia cases carrying *gelE*, were the first in Nigeria to produce *bla*_KPC_ and *oxa-48* carbapenemase genes. **Conclusions**: This study revealed the emergence of carbapenemase-producing *Enterococcus* species in our environment. A one-health approach and further molecular studies are essential to mitigate the spread and understand the transmission dynamics.

## 1. Introduction

*Enterococcus* species are non-spore-forming, facultative anaerobes that tolerate various environmental conditions [1]. This adaptability has made the organism part of the commensal flora of almost all terrestrial animals, including mammals, birds, reptiles, insects, farm animals, soil, plants, and aquatic ecosystems [2]. *E. faecium* and *E. faecalis* are the two most prevalent *Enterococcus* commensals in the gastrointestinal and genitourinary tracts, oral cavity, vagina, and skin, posing a significant public health concern to human and animal populations [3]. The intensity of antimicrobial use for different animal species has been reported to be among the crucial factors determining the levels of commensal enterococci resistance [4]. Antimicrobial resistance (AMR) bacteria are known to spread to humans through direct or indirect contact with animals or the consumption of animal products. Resistance genes from commensal *Enterococcus* strains in food animals may also transfer to human pathogenic bacteria over time, entering the environment and ecosystems through the use of manure as a fertilizer or via wastewater treatment discharges [5]. The diverse reservoir hosts, such as cattle, sheep, and goats, act as significant bio-indicators of environmental pollution with resistant bacteria from various sources [5]. The rise in AMR has been exacerbated by limited antibiotic discovery and inappropriate use (misuse and overuse) of available antibiotics, affecting different classes of drugs [6]. β-lactam drugs constitute roughly 65% of all antibiotics used globally for bacterial infections, encompassing penicillin, cephalosporins, monobactams, and carbapenems [6]. Notably, *E. faecalis* ranks as the second most commonly detected vancomycin-resistant enterococci (VRE) [7]. In Africa, antibiotics are often used as growth enhancers in livestock, poultry, and aquaculture, and they are commonly added directly to dairy products to prolong their shelf-life [8]. However, significant levels of antimicrobial drug residues in meat intended for human consumption pose a considerable risk for the spread and transmission of AMR bacteria [9]. In Sub-Saharan Africa, antibiotic resistance traits and antibiotic-resistant bacteria are normally transferred to the general public via food, the environment, and farm workers [10]. In Ghana, AMR *Enterococcus* spp. in livestock and raw meat samples, carrying multiple resistance (*tet(M), aph(3′)-III, lsa(A), erm(B), and lnu(B)*) and virulence (*ebpA, ebpB, cylB, hylB,* and *srB*) genes, including known clones associated with hospital-acquired infections, has been documented [11]. Also, in Nigeria, the transmission of this pathogen through the food chain has contributed to the spread of AMR (*tetK*, *tetL, tetM*, *tetO,* and ermB) and virulence (*asa1*, *gelE*, *and cylA*) genes [12]. Currently, carbapenems are highly effective against numerous bacterial species. They are less susceptible to many β-lactam resistance mechanisms, making them the most dependable last-resort antibiotics for treating bacterial infections. Carbapenems possess a characteristic β-lactam ring structure, which allows them to function by attaching to and deactivating penicillin-binding proteins (PBPs), essential components for constructing the bacterial cell wall [13]. This mechanism of action is a key factor in the potency of carbapenems as antibiotics [14]. Additionally, they have fewer side effects, making them a safer option than other last-line medications [15]. Furthermore, the distinctive molecular structure of carbapenems, which includes a carbapenem linked to a β-lactam ring, provides them with remarkable resistance against many β-lactamase enzymes [16]. Despite *Enterococcus* species exhibiting intrinsic resistance to many β-lactam antibiotics, carbapenems are an exception within the β-lactam class. Carbapenems are highly effective against Gram-positive and Gram-negative bacteria and offer a broad spectrum of antibacterial activity compared to other β-lactam antibiotics [15]. The emergence and dissemination of carbapenemase-producing bacteria, such as *Acinetobacter baumannii*, *Klebsiella pneumoniae*, and *Pseudomonas aeruginosa,* have raised substantial concerns due to their ability to infect humans and animals (both companion and food-producing) and their presence in environmental reservoirs [17]. The most prevalent carbapenemase genes are *bla*_IMI/NMC-A,_ *bla*_KPC,_ *bla*_VIM,_ *bla*_IMP,_ and *bla*_OXA-48_ [17]. However, carbapenem antibiotics are the most effective antimicrobials for treating infections caused by *E. faecium* [18]. Therefore, documented reports on carbapenemase-producing *Enterococcus* species in Africa, including Sub-Saharan countries such as Nigeria, are rare. This study investigated antimicrobial resistance patterns and carbapenemase-producing *Enterococcus* species in Lagos, Nigeria, from diverse sources.

## 2. Results

**Figure 1:**
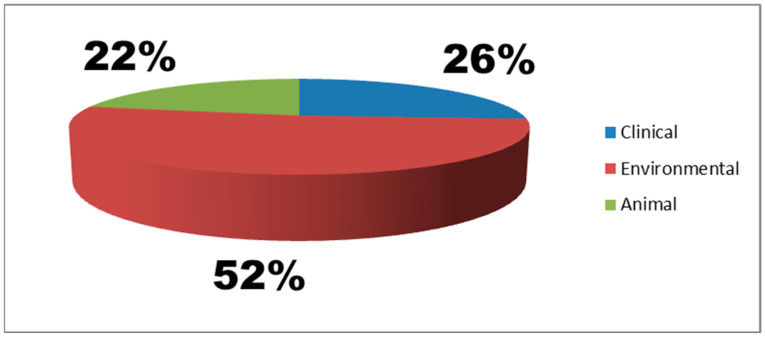
Occurrence of *Enterococcus* species isolated from clinical, animal, and environmental sources.

In this study, 23 *Enterococcus* isolates including 18 *E. faecalis* and 5 *E. faecium* were identified, with an overall prevalence of 4.6% (23/500) and source prevalences of 24% (12/50), 5% (5/100), and 1.7% (6/350) from environmental, animal, and clinical samples, respectively. Of the 12 environmental isolates, two strains of *E. faecium* were isolated from the abattoir effluent and 10 *E. faecalis* from both the abattoir effluent and the lagoon. Regarding the animal isolates, three strains of *E. faecium* and two *E. faecalis* were obtained from ready-to-slaughter cattle, while all six clinical isolates were *E. faecalis* in this study (Table 1 and Figure 1). However, there was no statistically significant relationship (*p* > 0.05) between the prevalence of positive *Enterococcus* spp. and the types of samples analyzed, with a significance level of 0.23. *E. faecalis* strains were isolated more in females than males, and the age group 19–37 was mainly implicated in this study. A statistically significant difference between age distribution and gender in the positive *E. faecalis* (*p* < 0.05) was recorded (Table 1). The antimicrobial susceptibility test (AST) results revealed that all *Enterococcus* isolates exhibited 100% resistance to ciprofloxacin, erythromycin, imipenem, vancomycin, meropenem, and ampicillin. High resistance levels were also noted for amoxicillin–clavulanic acid 95.7% (22/23), chloramphenicol 87% (20/23), trimethoprim–sulfamethoxazole 78.3% (18/23), and tigecycline 60.9% (14/23), while gentamicin had a lower resistance rate of 8.7% (2/23) (Table 2). *E. faecalis* and *E. faecium* isolates from clinical, environmental, and animal sources exhibited 82.6% (19/23) multidrug resistance (MDR). However, 39.1% (9/23) of MDR isolates carried the *gelE* virulence gene, mostly from environmental and human sources. Carbapenemase genes (*bla_KPC_* and *bla_OXA-48_*) identified in two *E. faecalis* strains isolated from human blood (HB003 and HB050) were found in patients with cases of bacteremia. Six resistance profiles (A-F) were observed, with pattern E (AMP-ERY-TGC-CIP-TS-VA-CHL-AUG-MEM-FOX-IMI) being the most frequent in *E. faecalis* from multiple sources. Additionally, unique resistance patterns were noted in a few *E. faecalis* and *E. faecium* isolates from animals (Table 3)

## 3. Discussion

Enterococci, a component of the normal intestinal flora in humans and animals, have become increasingly recognized as pathogens acquired within the community and are a significant cause of nosocomial infections [19]. In this study, the prevalence of *E. faecalis* from clinical settings is 1.7%, and the source prevalence of this pathogen in urine and blood samples is 1.4% and 0.3%, respectively. No *E. faecium* was isolated from clinical samples of human origin. This result contrasts with the 82.2% and 17.8% prevalence of *E. faecalis* and *E. faecium* reported in clinical samples, respectively, in Italy [20]. Several studies have documented disparate prevalence rates of *E. faecalis* in clinical specimens across diverse geographical regions. For example, investigations conducted in Pakistan, Tanzania, and Malaysia have reported prevalence rates of 20.8% [21], 15.3% [22], and 11% [23], respectively. The observed discrepancies between our findings and those of previous studies may be attributable to variations in study populations, geographic locations, sample sizes, sample types, isolation techniques, and antibiotic usage patterns [24]. Similar factors have been reported to influence the results of bacterial cultures from clinical samples [25]. Thus, the prevalence of *E. faecalis* indicates the spread of *Enterococci*-associated infections in both the community and hospital environment. Interestingly, 1.4% of females exhibited a higher prevalence of *E. faecalis* than the male subjects in this study. The increasing vulnerability and spread of infections by the female gender has been partly attributed to the proximity of the urethral opening to the bacterially rich vagina and rectum and its shorter distance from the bladder [26]. Additionally, 50% of *E. faecalis* strains from clinical human samples were isolated from the age group 19–37, making this group at risk of Enterococcal infections. In Winnipeg, Canada, the age group ≥65 years was reported to be at high risk of *Enterococcus*-associated infections in both women and men [27], and such infections were uncommon in men under 60. However, at age 80, men and women exhibit similar rates [27]. There is no statistically significant difference between age group and gender (*p* > 0.05) in the positive *E*. *faecalis* in clinical samples in this study. This result is at variance with a report from Brazil in which a variation was recorded in the Enterococcal prevalence between adolescents, adults, and gender [28]. Moreover, 12 (24%) *Enterococcus* isolates were recorded from the environmental samples. The increased prevalence of *Enterococcus* species in the environment can be attributed to contamination from water bodies and effluent, which are influenced by human activities, sewage discharge, and animal feces. The implication of this is the increasing spread of community-associated Enterococcal infections. These findings are consistent with a previous report that detected 63 *Enterococcus* species in the final effluent of wastewater treatment plants and downstream environmental waters [29]. This affirms the significance of the *Enterococcus* species as a reliable indicator for evaluating water quality.

Furthermore, the prevalence of 5% *Enterococcus* species was recorded in animal samples. This result is low compared to the 42.8% prevalence reported in a study conducted in Nigeria on cattle and poultry animals [12]. A 34% prevalence was recorded in a study conducted in Egypt on the milk of cattle and buffalo [30]. The variation could be attributed to host-related factors, such as diet, which can influence the composition of commensal bacteria in the animal gut [30]. This may account for the observed differences in the distribution of *Enterococcus* species recorded. The detection of *E. faecalis* and *E. faecium* in animal samples holds significance, as these species are the most predominant among Enterococci-causing human infections [3] and could be a major contributor to animal mortality. The consequence of this finding is the possibility of continuous zoonotic transmission of *Enterococcus* species in our environment.

In Nigeria, antibiotic use both in animal and human medicine, and factors such as uncontrolled use of antibiotics in the form of over-the-counter purchase and street hawking of drugs, all have a role in the emergence, persistence, and spread of antibiotic resistance (Akinyemi and Fakorede, 2018) [31]. Also, the improper use of antibiotics usually leaves antibiotic residues in animal-derived products and the environment, thus heightening the risk of resistant bacterial transmission along the food chain (Ayukekbong et al., 2016) [9]. The result of the antibiotic susceptibility test conducted consistently indicated that 100% of *Enterococcus* species were resistant to several antibiotics, namely ciprofloxacin, erythromycin, imipenem, vancomycin, meropenem, and ampicillin. Also, all the 23 *Enterococcus* isolates unveiled resistance to vancomycin. This result is consistent with the findings from South Africa, where over 95% of the *Enterococcus* species were vancomycin-resistant [32], and in Iran, where vancomycin-resistant *Enterococcus* species were high in children [33]. In contrast, a study reported a low resistance rate (3.3%) to these antibiotics in Turkey [34].

Furthermore, the study also revealed 100% resistance to ampicillin. This resistance rate is relatively higher than the 46% reported from Jimma, Ethiopia [23]. Interestingly, there was also a high resistance rate to chloramphenicol and tigecycline, which aligns with a study from Tanzania [22] and is in contrast to a study conducted in Kenya, where *Enterococcus* species were 100% susceptible to tigecycline [35]. It was observed that *Enterococcus* isolates were 100% resistant to carbapenem antibiotics (imipenem and meropenem). Carbapenems are potent antibiotics used primarily for multidrug-resistant (MDR) infections. Although these antibiotics are often unavailable in hospitals in developed African countries due to high costs, studies reveal the emergence of carbapenem-resistant bacteria (CRB) in Sub-Saharan Africa (SSA) [36,37]. This result corroborated a study conducted in Denmark in 2007, where two of the isolates from the blood of patients with bacteremia were resistant to imipenem [38]. Furthermore, this study revealed a high resistance rate of *E. faecalis* and *E. faecium* to ciprofloxacin. Similar reported cases of ciprofloxacin resistance in *E. faecalis* and *E. faecium* from poultry occurred in Korea [39]. All *Enterococcus* isolates were MDR. They developed resistance to different antimicrobials from three to five distinct classes.

The high prevalence of MDR isolates observed in this study reflects the widespread use of broad-spectrum antibiotics. These findings align with other research conducted in Nigeria, which indicates that *E. faecalis* and *E. faecium* present significant concerns due to their ability to develop resistance to various antibiotics, including vancomycin and other commonly used therapeutic agents [40,41]. Interestingly, the observed MDR pattern is consistent with a study in India involving *Enterococcus* species isolated from animals, humans, and food sources [42]. These MDR strains pose a significant public health risk, particularly considering that the same class of antibiotics is commonly used to treat various bacterial infections in humans and animals. Interestingly, there was a high susceptibility to gentamicin; only 8.7% of the isolates developed resistance. This finding is slightly similar to the lower prevalence of 21% *E. faecalis* and 35% *E. faecium* isolates that developed resistance to gentamicin in Iran [43]. This situation might be associated with the judicious use of gentamicin antibiotics, which prevent the development and spread of resistant bacteria. This study suggests that gentamicin could be used instead of vancomycin as an alternative antibiotic for patients with *Enterococcus*-associated diseases. However, Enterococci have restricted permeability to aminoglycosides, and the antibiotics needed to eradicate the bacteria effectively are often too high to administer to humans safely [44]. In this study, all *Enterococcus* isolates exhibited six resistance patterns with pattern E, with AMP-ERY-TGC-CIP-TS-VA-CHL-AUG-MEM-IMI being the most frequently encountered in 47% of the *Enterococcus* species isolated. In this study, 41.7% of Enterococcal isolates from the environment harbored a virulence gene (*gelE*). This virulence determinant (*gelE*) produced by *E. faecalis* is known to degrade host proteins and weaken immune defenses through complement inactivation, thus aiding bacterial spread and survival [45]. This gene may influence its pathogenicity, biofilm formation, and antibiotic resistance, making it a crucial factor in clinical infections and environmental persistence. Nonetheless, 43.8% (7/16) of *E. faecalis* strains from clinical and environmental isolates were found to carry the *gelE* virulence gene.

Additionally, 66.7% (2/3) of *E. faecium* strains from animal isolates exhibited this gene. This percentage is lower than the 84% reported in Cairo, Egypt, from clinical isolates [23,46]. This variation in prevalence rates may be partly attributed to differences in the distribution of virulent strains in different geographical areas. Notably, the transfer of virulence genes is interconnected among the sources. It can play a role in the emergence and spread of bacterial antibiotic resistance, affecting human populations, animals, and the environment [3,46].

Interestingly, the *E. faecalis* strains HB003 and HB050 carrying the virulence gene *gelE*, isolated from cases of bacteremia, were found to produce carbapenemase genes *bla_KPC_* and *oxa-48.* This finding was further corroborated by a report from Munita and Arias [47], which showed that *Enterococcus* species can acquire carbapenemase genes, such as *bla_KPC_, bla_NDM,_ bla_OXA_,* and *bla_VIM_*_,_ through horizontal gene transfer. Notwithstanding, these mechanisms have the unintended consequence of perpetuating the multidrug resistance crisis by facilitating the rapid dissemination of resistance genes, not only within *Enterococcus* strains but also across interspecies boundaries [48]. This phenomenon has profound implications for clinical practice, as it severely constrains therapeutic options and presents a formidable challenge in the management of infectious diseases in clinical settings [48]. The detection of these gene markers in *E. faecalis strains* indicates the potential for cross-species transmission and the emergence of carbapenemase-producing *E. faecalis* [3,48]. These findings highlight the challenges in treating infections caused by *Enterococcus* strains that harbor resistance genes and emphasize the importance of infection control measures in humans and animals [48]. Moreover, this study revealed the dominance of *E. faecalis* strains as vectors of carbapenemase genes (*bla*_KPC_ and *blaoxa*-_48_) in Nigerian clinical settings, a previously unreported phenomenon. This finding has significant implications for the management of Enterococcus-associated infections, potentially limiting the therapeutic options. The study’s limitations include a modest sample size and geographically restricted focus on Lagos, Nigeria, which may compromise generalizability. Future research should aim to include larger, more diverse populations and employ advanced methodologies, such as whole-genome sequencing, to elucidate the evolutionary dynamics of these resistance-resistant genes.

## 4. Materials and Methods

### 4.1. Study Design, Place, and Duration of Study

A cross-sectional study was conducted from November 2023 to February 2024 involving 500 samples: 350 from patients at Lagos State University Teaching Hospital (LASUTH) and Badagry General Hospital (BGH), 50 environmental samples (effluent) from Maza-Maza lagoon and Oto-Awori, and 100 cow rectal and nasal swab samples from Oko-Oba and Oto-Awori abattoirs.

The study included clinical samples primarily from patients at the two main referral hospitals (LASUTH and BGH), focusing on those with specific conditions such as chronic orthopedic and post-surgical wound infections, urinary tract infections, chronic skin infections, chronic respiratory diseases, ventilator-associated pneumonia, and cardio-pulmonary diseases. The patients cut across all age groups and had been hospitalized for at least two weeks. Environmental samples were collected exclusively from Maza-Maza lagoon and Oto-Awori. Samples of animal origin were collected only from rectal and nasal swabs of cattle.

The study excluded clinical samples from outpatients and those who did not exhibit the symptoms specified in the inclusion criteria. Patients with a hospital stay of less than two weeks were also excluded. Environmental samples were collected from areas outside the designated study area, and food-animal samples not collected from the cloaca/rectal area, particularly those not within the study area, were not included in this study.

### 4.2. Sample Size and Sampling

The sample size for the clinical samples was obtained using the following formula

n = (Z^2^ × P × q)/d^2^

where P = prevalence of the previous study, 73% = 0.73 [41].
where n= number of samples to be collected.
where q = 1 − P (proportion of the population without the characteristic).
Z = confidence level at 95% (standard value of 1.96).
d = margin of error at 5% (standard value of 0.05).
P = prevalence rate.
q = 1 − P = 1 − 0.73 = 0.27.
d = allowable error = 5%.
Z = standard normal distribution at 95% CI = 1.96.
n = (1.96^2^ × 0.059 × 0.27)/0.05^2^.
n = 65.

However, 350 clinical samples were collected from the hospitals used, comprising 150 blood samples, 100 urine samples, 50 wound swabs, and 50 sputum samples. Specifically, the 100 animal samples consisted of 50 cattle rectal swabs and 50 nasal swabs. Also, 50 environmental samples were made up of 25 abattoir effluent and Lagoon water samples.

### 4.3. Bacterial Isolation and Identification 

Approximately 5 mL blood samples were collected from patients exhibiting clinical symptoms of chronic septicemia and placed in a sterile container with 50 mL of thioglycolate broth, using a blood-to-broth ratio of 1:10. Blood culture bottles were incubated at 37 °C and examined daily after 48 h for five consecutive days. The presence of turbidity, hemolysis, gas formation, or color changes indicated microbial growth. If no growth was observed within 7 days, the result was reported as negative. When visible growth appeared, a small quantity of broth was aseptically taken and subcultured on Bile-esculin azide agar (BEAA) (Tintan media, Rajasthan, India) from Hardy Diagnostics. Urine samples were inoculated on BEAA media using a 10 mL calibrated loop and incubated at 37 °C for 24 h. The presence of 10^4^ colony-forming units per mL of bacteria with black-colored colonies indicated significant Enterococci in the urine. Other clinical samples were directly inoculated on BEAA and incubated at 37 °C for 24 h, checking for the growth of black-colored colonies.

The membrane filtration methods were used to isolate *Enterococcus* species from environmental water and effluent samples [49]. One-hundred mL of water were filtered through a 0.45 μm-sized membrane filter. The water flow was facilitated using an air vacuum pump attached to a conical flask to collect the unwanted filtered water. The filter paper was placed on the surface of differential media BEAA (Tintan media, Rajasthan, India), plates were incubated at 37 °C for 24 h, and colonies were selected according to their shape, size, and color.

Animal samples were introduced into Todd Hewitt broth and incubated at 37 °C for 24 h under aerobic conditions. Following this, the broth was cultured onto BEAA (Tintan media, Rajasthan, India) and subjected to aerobic incubation at 37 °C for an additional 24 h [50]. Subsequently, the visible colonies were visually inspected for color, size, and shape. Confirmation of Enterococci presence involved additional tests such as Gram stain, catalase reaction, growth on broth containing 6.5% NaCl, and growth in BHI broth at 37 °C and 45 °C for 48 h [51].

### 4.4. Antimicrobial Susceptibility Testing (AST)

The antimicrobial susceptibility of the isolates was determined using the Kirby–Bauer disk-diffusion method. Antibiotic discs (Mast Diagnostics, Merseyside, UK) were dispensed onto Muller–Hinton agar using an automated disc dispenser. The following antibiotic discs were used: clindamycin (2 μg), imipenem (10 μg), ciprofloxacin (15 μg), vancomycin (30 μg), amoxicillin–clavulanic acid (10 μg), cefoxitin (30 μg), ampicillin (10 μg), ceftriaxone (30 μg), ceftazidime (30 μg), meropenem (10 μg), and gentamycin (30 μg). Strains showing intermediate resistance were considered susceptible dose dependent according to the Clinical and Laboratory Standards Institute (CLSI) guidelines [52]. Where VET01S breakpoints were unavailable, human breakpoints were applied. The diameter zones of inhibition were interpreted as the performance standards outlined in the guideline. The control strain used for testing was *E. coli* ATCC 25922. Multidrug resistance (MDR) in this study was defined as resistance to 3 or more antimicrobial classes. The resistance index was calculated as the ratio of the number of antibiotics resistant to the total number of antibiotics used.

### 4.5. Phenotypic Detection of Carbapenemase-Producing Enterococcus Species

Enterococcus species that developed resistance to imipenem and meropenem according to CLSI guidelines [52] were subjected to the Modified Hodge test [53]. In brief, a 0.5 McFarland standard of *E*. *coli* ATCC 25,922 suspensions were prepared in 5 mL of saline. A 1:10 dilution (0.5 mL of suspension at 4.5 mL of saline) was streaked as a lawn onto Muller–Hinton Agar (MHA) plate and dried for about 3–10 min. A disc of 10 mcg meropenem was placed at the center of the inoculated MHA plate. The test organism was streaked in a straight line from the edge of the disc to the edge of the plate. It was incubated at 37 °C for 24 h. An enhanced growth formation of a clover leaf-like pattern indicates a positive carbapenemase production [53].

### 4.6. DNA Extraction, Resistance, and Virulence Gene Detection

DNA was extracted from bacterial colonies grown on bile–esculin agar using bacterial lysis buffer and heat treatment. These were carried out using the Luna Universal qPCR protocol (New England BioLabs), following the manufacturer’s instructions. The process involved mechanical disruption using a bead beater for 25 min, followed by centrifugation of the ZR Bashing Bead Lysis Tube at 10,000× *g* for 1 min. β-Mercaptoethanol was added to the Genomic Lysis Buffer (final dilution: 0.5%) for enhanced performance. Pure Enterococcus isolate cells were placed into the lysis tube with the Bashing Bead Buffer and centrifuged at high speed for 15 min. The supernatant was filtered through a Zymo-Spin II-F Filter (Zymo Research, Irvine, CA, USA, 8000× *g*, 1 min) and underwent several washing and centrifugation steps with a Zymo-Spin ICR Column. The DNA was then eluted by adding DNA Elution Buffer to the column and centrifuging at 10,000× *g* for 3 s, resulting in ultra-pure DNA. The supernatant containing the extracted DNA was collected and stored at −20 °C. The RT-PCR Rotor-Gene Q 2plex (Qiagen, Hilden, Germany) amplification was performed using specific primer sets for resistance (*bla_SHV,_ bla_TEM,_ bla_KPC,_ blaoxa-48,* and *blaImp*) and virulence (*esp and* gelE) genes (Table 4), with PCR mixtures prepared using specific volumes and concentrations. The PCR process entailed 45 cycles, each consisting of a denaturation phase at 95 °C for 30 s, followed by an annealing step at 50 °C for 30 s, and concluding with an extension at 72 °C for 60 s [54].

### 4.7. Statistical Analysis

In this study, the data collected were input into a computer and analyzed using IBM SPSS version 20. Frequency tables were created, and the relationships between the variables were analyzed using the Chi-square test or *t*-test, with a significance level set at *p* < 0.05.

## 5. Conclusions

This study revealed the prevalence of MDR and the emergence of carbapenemase-producing *Enterococcus* species in our environment. It also revealed *E. faecalis* strains carrying carbapenemase genes *bla_KPC_* and *oxa-48* from bacteremia cases. *Enterococcus* isolates were found to exhibit heterogeneous resistance profiles. Gentamicin antibiotic was 91% effective on *Enterococcus* isolates and may be used for the treatment of *Enterococcus*-associated diseases when indicated. The findings indicated the significance of screening for antibiotic resistance among *Enterococcus* from diverse sources. Further investigation into their resistance mechanisms and origins from a One Health perspective is essential.

## Figures and Tables

**Table 1:** Prevalence and distribution of *Enterococcus* species isolated from different sources.

Category	Sub-Category	Parameters	No. of Samples	No. of + Samples	*Enterococcus* spp. Isolated	
					*E. faecalis*	*E. faecium*
Clinical	Hospital	BGH	175	2	2	0
		LASUTH	175	4	4	0
		**Total**	**350**	**6**	**6**	**0**
	Samples collected	Urine	100	4	4	0
		Blood	150	2	2	0
		Sputum	50	0	0	0
		Wound	50	0	0	0
		**Total**	**350**	**6**	**6**	**0**
	Sex	Male	127	1	1	0
		Female	223	5	5	0
		**Total**	**350**	**6**	**6**	**0**
	Age	0–18	100	1	1	0
		19–37	150	3	3	0
		38–56	50	0	**0**	0
		>57	50	2	2	0
		**Total**	**350**	**6**	**6**	**0**
Animal	Study area	Oko-Oba abattoir	50	1	1	0
		Oto Awori abattoir	50	4	1	3
	Samples collected	Cattle Nasal swab	50	1	1	0
		Cattle Rectal swab	50	4	1	3
		**Total**	**100**	**5**	**2**	**3**
Environmental	Effluent	Oko-Oba abattoir	25	5	3	2
	Lagoon water	Maza-Maza	25	7	7	0
		**Total**	**50**	**12**	**10**	**2**
		**Ground Total**	**500**	**23**	**18**	**5**

+ = positive.

**Table 2:** Antibiotic susceptibility, resistance, and virulence genes of *Enterococcus* species from different sources.

			Antibiotics Susceptibility											Strain Code	No. of Resistance	No. of Sensitive	^3^ RI	Suspected Organism
Sample Source	Sample Type	Sample Location	^1^ GM	^1^ AMP	^1^ ERY	^1^ TGC	^1^ CIP	^1^ TS	^1^ VA	^1^ CHL	^1^ AUG	^1^ MEM	^1^ IMI					
Clinical	Blood	Badagry	S	R	R	R	R	R	R	R	R	R	R	^2^ HB003	10	1	0.1	*E. faecalis*
			S	R	R	S	R	R	R	R	R	R	R	^2^ HB050	10	1	0.1	*E. faecalis*
	Urine	LASUTH	S	R	R	R	R	R	R	R	R	R	R	^2^ HU052	10	1	0.1	*E. faecalis*
			S	R	R	R	R	R	R	R	R	R	R	^2^ HU062	10	1	0.1	*E. faecalis*
			S	R	R	S	R	R	R	R	R	R	R	^2^ HU074	9	2	0.2	*E. faecalis*
			S	R	R	R	R	R	R	R	R	R	R	^2^ HU075	10	1	0.1	*E. faecalis*
Animal	Nasal Swab	Oko-Oba	S	R	R	R	R	S	R	R	R	R	R	^2^ ANS1	9	2	0.2	*E. faecalis*
	Rectal Swab	Oto-Awori	S	R	R	S	R	R	R	R	R	R	R	^2^ ARS20	9	2	0.2	*E. faecium*
			R	R	R	S	R	R	R	R	R	R	R	^2^ ARS21	10	1	0.1	*E. faecium*
			S	R	R	R	R	R	R	R	R	R	R	^2^ ARS22	10	1	0.1	*E. faecalis*
			R	R	R	R	R	R	R	R	R	R	R	^2^ ARS23	11	0	0	*E. faecium*
Environmental	Lagoon Water	Maza-Maza	S	R	R	S	R	S	R	S	R	R	R	^2^ EL1	7	4	0.6	*E. faecalis*
			S	R	R	R	R	R	R	R	R	R	R	^2^ EL12	10	1	0.1	*E. faecium*
			S	R	R	S	R	S	R	S	R	R	R	^2^ EL14	10	1	0.1	*E. faecium*
			S	R	R	R	R	R	R	R	R	R	R	^2^ EL15	10	1	0.1	*E. faecalis*
			S	R	R	R	R	R	R	R	R	R	R	^2^ EL17	10	1	0.1	*E. faecalis*
			S	R	R	R	R	R	R	R	R	R	R	^2^ EL21	10	1	0.1	*E. faecalis*
			S	R	R	R	R	R	R	R	R	R	R	^2^ EL5	10	1	0.1	*E. faecalis*
	Effluent	Oko-Oba	S	R	R	R	R	R	R	R	R	R	R	^2^ EE2	10	1	0.1	*E. faecalis*
			S	R	R	S	R	S	R	S	R	R	R	^2^ EE3	7	4	0.6	*E. faecalis*
			S	R	R	S	R	S	R	R	S	R	R	^2^ EE 5	7	4	0.6	*E. faecalis*
			S	R	R	S	R	R	R	R	R	R	R	^2^ EE 6	9	2	0.2	*E. faecalis*
			S	R	R	R	R	R	R	R	R	R	R	^2^ EE 8	10	1	0.1	*E. faecalis*

^1^ Antibiotics: AMP, ampicillin; AUG amoxicillin–clavulanic acid; GM, gentamycin; CIP, ciprofloxacin; ERY, erythromycin; IMI, imipenem; VA, vancomycin; CHL, chloramphenicol; TGC, tigecycline; MEM, meropenem; TS trimethoprim sulfamethoxazole; ^2^ Samples: HB, human blood; HU, human urine; ANS, animal nasal swab; ARS, animal rectal swab; EL, lagoon water; EE, environmental effluent; ^3^ Resistance index (RI): number of antibiotics resisted/total number of antibiotics used.

**Table 3:** Resistance pattern, phenotypic, and genotypic detected resistance and virulence genes found in *Enterococcus* isolates.

Resistance Patterns	Pattern Code	No of Isolates	No. of Antibiotics Resisted	*E. faecalis*			*E. faecium*		
				Strain Code	Resistance Gene (Code)	Virulence Gene (Code)	Strain Code	Resistance Gene (Code)	Virulence Gene (Code)
AMP-ERY-TGC-CIP-VA-CHL-AUG-MEM-IMI	A	1	9	ANS1	-	-	-	-	-
AMP-ERY-CIP-VA-AUG-MEM-IMI	B	3	7	EL14, ELI	-	*gelE* (EL1)	EE3	-	-
GM-AMP-ERY-CIP-TS-VA-CHL-AUG MEM-IMI	C	1	10	-	-	-	ARS21	-	*gelE* (ARS21)
AMP-ERY-TGC-CIP-TS-VA-CHL-AUG-MEM-IMI	D	3	10	HB050, EE6, EE8	*bla_OXA-48_*, bla*_KPC_*, (B050)	*gelE* (HB050)	-	-	-
AMP-ERY-TGC-CIP-TS-VA-CHL-AUG-MEM-IMI	E	11	10	HU052, HU062, HU075, EL12, HB003, EL15, EL17, EL21, EL5 EE2	*bla_OXA-48_*, bla*_KPC_*, (HB003)	*gelE* (HB003, EL5, EL15, EL17, EL21)	EE5	-	-
GM-AMP-CO-TGC-CIP-TS-VA-CHL-AUG--MEM-IMI	F	1	11	-	-	-	ARS23	-	*gelE* (ARS23)

Keys: AMP, ampicillin, AUG, amoxicillin–clavulanic acid: GM, gentamycin; CIP, ciprofloxacin; ERY, erythromycin, IMI, imipenem; VA, vancomycin; CHL, chloramphenicol; TGC, tigecycline; MEM, meropenem; TS, trimethoprim–sulfamethoxazole; HB, human blood; HU, human urine; ANS, animal nasal swab; ARS, animal rectal swab; EL, lagoon water; EE, environmental effluent.

**Table 4:** Primers for molecular identification of resistance and virulence genes.

S/N	Genes	Primer Sequence 5′-3′	Size (bp)	Reference
1	*bla_SHV_*,	F-3′CGCCTGTGTATTATCTCCCT’ R-5′CGAGTAGTCCACCAGATCCT’	293	[55]
2	*bla_TEM_*,	F-3′TTTCGTGTCGCCCTTATTCC R-5′ATCGTTGTCAGAAGTAAGTTGG	403	[55]
3	*bla_KPC_*	F-3′CGTCTAGTTCTGCTGTCTTG R-5′CTTGTCATCCTTGTTAGGCG	798	[56]
4	*blaoxa-48*	F-3′GCGTGGTTAAGGATGAACAC R-5′CATCAAGTTCAACCCAACCG’	550	[57]
5	*blaImp*	F-3′GGAATAGAGTGGTTAAYTCTC R-5′GGTTTAAYAAAACAACCACC’	232	[58]
6	*esp*	F AATTGATTCTTTAGCATCTGG R AGATTCATCTTTGATTCTTGG	510	[59]
7	*gelE*	F-TATGACAATGCTTTTTGGGAT R-AGATGCACCCGAAATAATATA	213	[59]

## Data Availability

The original contributions presented in this study are included in the article. Further inquiries can be directed to the corresponding authors.
